# Trends in sleep medication prescriptions and the incidence of falls, delirium, and self‐removal of drains and tubes over a 3‐year period among 15,721 patients, including those with dementia, at Kyushu University Hospital

**DOI:** 10.1002/alz70857_099223

**Published:** 2025-12-24

**Authors:** Toshifumi Minohara, Kaishi Takabatake, Taro Nakazawa, Tomoyuki Ohara, Tomohiro Nakao

**Affiliations:** ^1^ Graduate School of Medical Sciences, Kyushu University, Fukuoka, Fukuoka, Japan

## Abstract

**Background:**

To investigate sleep medication prescriptions over a 3‐year period for all patients including those with dementia who admitted to Kyushu University Hospital, as well as trends in incident reports, such as fall and delirium, in the hospital over the same period.

**Method:**

This study included 15,721 patients (22,229 prescriptions) who were hospitalized at Kyushu University Hospital (Fukuoka, Japan) between April 1, 2019, and March 31, 2021, and were prescribed sleep medication during their hospital stay. Data on prescribed sleep medications, dementia status, and psychiatric liaison interventions were extracted from medical records. Additionally, information on falls, delirium, and self‐removal of drains and tubes was collected from in‐hospital incident reports. The study was approved by the Ethics Committee of the Faculty of Medical Sciences, Kyushu University.

**Result:**

Among all hospitalized patients, the proportion of GABA receptor agonist prescriptions decreased from 54% in 2019 to 37% in 2021, while the proportion of orexin receptor antagonist prescriptions increased from 34% to 52% over the same period. Similar trends were observed in a subgroup analysis of patients who received psychiatric liaison team interventions, including those with dementia, where the proportion of orexin receptor antagonist prescriptions increased from 48% to 65%. During this period, the number of in‐hospital incidents, such as delirium, falls, and self‐removal of drains and tubes, showed a decreasing trend that inversely correlated with the increased prescription of orexin receptor antagonists. Notably, the number of incidents involving delirium was reduced by approximately half.

**Conclusion:**

This study demonstrated that a shift in sleep medication prescriptions from GABA receptor agonists to orexin receptor antagonists were observed among 15,721 hospitalized patients, even in general medical departments. The observed decreasing trend in the number of incidents, such as falls and delirium in patients with dementia, suggests that changes in hypnotic prescription patterns may have contributed to these improvements.

